# CDK4/6 inhibition in advanced chordoma: final results of the NCT PMO-1601 trial

**DOI:** 10.1016/j.esmoop.2025.105498

**Published:** 2025-07-07

**Authors:** M.-V. Teleanu, C.E. Heilig, S. Pirmann, R. Hamacher, S. Bauer, V.I. Gaidzik, R. Mayer-Steinacker, J. Al-Sabah, S.S.L. Roldan Pinzon, H. Süße, A. Freitag, P. Horak, S. Kreutzfeldt, B. Hutter, J. Hüllein, O. Sedlaczek, B. Lehner, G. Egerer, D. Jäger, C. Müller-Tidow, D. Hübschmann, C. von Kalle, T.F.E. Barth, S. Fröhling, R.F. Schlenk

**Affiliations:** 1 Division of Translational Medical Oncology, National Center for Tumor Diseases (NCT) Heidelberg and German Cancer Research Center (DKFZ), Heidelberg, Germany; 2 Department of Hematology, Oncology and Rheumatology, Heidelberg University Hospital, Heidelberg, Germany; 3 German Cancer Consortium (DKTK), Core Center Heidelberg, Heidelberg, Germany; 4 Computational Oncology Group, Molecular Precision Oncology Program, German Cancer Research Center (DKFZ) and National Center for Tumor Diseases (NCT) Heidelberg, Heidelberg, Germany; 5 Department of Medical Oncology and Sarcoma Center, West German Cancer Center, University Hospital Essen, Essen, Germany; 6 German Cancer Consortium (DKTK), Partner Site University Hospital Essen, Essen, Germany; 7 Department of Internal Medicine III, Ulm University Hospital, Ulm, Germany; 8 NCT Trial Center, DKFZ and Heidelberg University Hospital, Heidelberg, Germany; 9 Divison of Radiology, German Cancer Research Center, Heidelberg, Germany; 10 Department of Orthopaedics, Heidelberg University Hospital, Heidelberg, Germany; 11 Department of Medical Oncology, NCT Heidelberg and Heidelberg University Hospital, Heidelberg, Germany; 12 Heidelberg Institute for Stem Cell Technology and Experimental Medicine (HI-STEM), Pattern Recognition and Digital Medicine Group, Heidelberg, Germany; 13 Innovation and Service Unit for Bioinformatics and Precision Medicine, German Cancer Research Center (DKFZ), Heidelberg, Germany; 14 Institute of Human Genetics, Heidelberg University, Heidelberg, Germany; 15 Berlin Institute of Health, Berlin, Germany; 16 Institute of Pathology, Ulm University Hospital, Ulm, Germany

**Keywords:** advanced chordoma, *CDKN2A*/p16 inactivation, CDK4/6 inhibition, palbociclib, multicenter phase II trial

## Abstract

**Background:**

This study aims to evaluate antitumor response of palbociclib in patients with advanced chordoma, an ultra-rare cancer without approved systemic therapy. Previous data showed that palbociclib reduced cell viability and proliferation in *CDKN2A*-deficient chordoma cell lines.

**Patients and methods:**

We conducted a phase II single-arm, open-labeled trial on palbociclib in adult patients with advanced chordomas with p16 (by immunohistochemistry) or *CDKN2A* (by genomic analysis) loss along with retained CDK4/6 and RB1 expression (by immunohistochemistry or RNA sequencing). Based on CDK4/6/pRB (S780) immunohistochemical expression patterns, a responder versus non-responder signature was assigned. The study used a Simon optimal two-stage design with the primary endpoint of disease control rate (DCR) by RECISTv1.1 after six cycles. Secondary endpoints included progression-free survival, overall survival, and biomarker analysis. The study was considered positive if 25% of patients reached the primary endpoint.

**Results:**

Twenty-eight patients with a median age of 59 years were assessed for the primary endpoint. After a median follow-up of 28 months, the DCR was 39%, with 11 patients achieving stable diseases. No objective responses were obtained. The median progression-free survival was 5.6 months, and the median overall survival was 24.6 months. Treatment was well tolerated without new safety signals. There was no correlation between immunohistochemical responder phenotypes and outcome. Biomarker analysis identified additional clinically actionable alterations affecting *PIK3CA, PTEN, MTAP,* or *MET* genes, and druggable pathways by transcriptomic analysis.

**Conclusion:**

Although antitumor activity was modest, the trial met its primary endpoint. Molecularly tailored combination therapies should be considered in the future to improve efficacy.

## Highlights

• Palbociclib monotherapy had modest activity in advanced p16-deficient chordoma; however, signals of activity were observed.
• Genomic and transcriptomic profiling can uncover further druggable alterations (e.g. *MTAP* loss, *PIK3CA* mutations).
• Molecularly guided treatment should be considered for all chordoma patients who are candidates for systemic therapy.
• Collaborative networks are essential to advance clinical research in ultra-rare cancers.

## Introduction

Chordoma is an ultra-rare bone tumor^1^ that arises from remnants of the embryonic notochord and affects the skull base, pelvis, and, less commonly, the mobile spine.^2^ Surgical resection and different radiotherapy strategies are the pillars of chordoma treatment. However, depending on the clinical features and treatment approaches, many patients will have local recurrence and up to 40% of patients will develop metastases.^3^^,^^4^ To date, there are no approved drugs for the treatment of locally advanced or metastatic chordoma. Conventional chemotherapeutics such as doxorubicin or ifosfamide have shown only limited clinical benefit, at least in part explained by the slow growth of most chordomas.^5^ In addition, several targeted drugs, such as imatinib, lapatinib, erlotinib, and sirolimus, directed against platelet-derived growth factor receptor alpha/beta (PDGFRA/B), epidermal growth factor receptor (EGFR), and mechanistic target of rapamycin complex 1, respectively, have been repurposed but yielded only modest response rates. Nevertheless, single cases of long-lasting disease stabilization have been reported.^6^, ^7^, ^8^, ^9^

At the molecular level, chordoma is a heterogeneous disease.^10^, ^11^, ^12^ However, only ∼14% of patients harbor alterations that can be targeted by United States Food and Drug Administration-approved drugs,^4^ and molecularly stratified clinical trials are mainly lacking. More than half of chordoma patients exhibit inactivation of the tumor suppressor p16 (cyclin-dependent kinase inhibitor 2a, encoded by *CDKN2A*), leading to constitutive activation of the cyclin D-cyclin-dependent kinase 4/6 (CDK4/6)-retinoblastoma (RB) pathway,^13^^,^^14^ which may represent an opportunity for therapeutic intervention. Palbociclib is a CDK4/6 inhibitor that blocks the transition from the G1 to the S phase of the cell cycle and has limited activity against other kinases.^15^^,^^16^ It is approved for the treatment of breast cancer in combination with endocrine therapy and has also been studied in CDK4-amplified liposarcomas^17^^,^^18^ and other solid tumors with deregulation of the CDK4/6-RB axis.^19^, ^20^, ^21^, ^22^ Consistent with the hypothesis that CDK4/6 may be therapeutic targets in chordoma, palbociclib inhibits the proliferation of p16-deficient chordoma cell lines, and a molecular signature predicting sensitivity to CDK4/6 blockade has been proposed that includes p16 loss and expression of CDK4/6 and phosphorylated RB1 (S780).^13^

To investigate the efficacy of palbociclib in patients with advanced chordoma, we conducted a multicenter phase II study as part of the National Center for Tumor Diseases (NCT) Precision Medicine in Oncology (PMO) program, NCT PMO-1601 (NCT03110744), which enrolled patients with p16 loss [by immunohistochemistry (IHC)] or *CDKN2A* loss (by genomic analysis) and CDK4/6 and RB1 presence (by IHC or RNA sequencing).

## Patients and methods

### Patient selection and treatment

Eligible patients were at least 18 years old and had locally advanced or metastatic chordoma not amenable to curative treatment by surgery or radiation. A confirmed progressive disease per RECISTv1.1 on the last treatment before enrollment was not mandatory. Key inclusion criteria included a molecular responder phenotype [defined as p16 or *CDKN2A* loss, as determined by IHC or genomic analysis, and CDK4/6 and RB1/pRB1S780 expression, as determined by IHC or transcriptomic] analysis, advanced or metastatic disease with at least one measurable lesion according to RECISTv.1.1, not amenable to curative treatment with surgery or radiotherapy. Further inclusion criteria included adequate organ function and Eastern Cooperative Oncology Group performance status ≤2 (Supplementary Information, available at https://doi.org/10.1016/j.esmoop.2025.105498). At study initiation, failure of prior treatment with a tyrosine kinase inhibitor, e.g. imatinib, was an inclusion criterion; however, based on the amendment of November 2020, this criterion was removed after 20 enrolled patients. Eligible patients received palbociclib 125 mg once daily, on days 1-21 of a 28-day cycle. All patients who received at least one palbociclib dose were included in the safety analysis. Treatment-related adverse events were defined as those occurring from the first palbociclib dose to 28 days after treatment discontinuation. Patients were treated with palbociclib until disease progression, unacceptable toxic effects, consent withdrawal, or the patient’s last visit, whichever occurred first.

The study was conducted in accordance with the Declaration of Helsinki and the International Conference on Harmonization Good Clinical Practice Guideline at three comprehensive cancer centers in Germany: NCT Heidelberg, West German Cancer Center Essen; Ulm University Hospital. The study protocol and amendment were approved by the competent authority and the leading independent ethics committee at Heidelberg University. The study was supervised by an Independent Data Monitoring Committee. All patients provided written informed consent before participation in the study.

### Study design and statistical analysis

NCT PMO-1601 was a non-randomized, single-arm, open-label, multicenter phase II trial designed to investigate the antitumor activity of palbociclib in adults with advanced chordoma.

The primary study endpoint was disease control rate (DCR) after six cycles of palbociclib, defined as complete response (CR), partial response (PR), or stable disease (SD) according to RECISTv1.1. Secondary endpoints included overall response rate, progression-free survival (PFS), and overall survival (OS). Exploratory endpoints were related to correlative biomarker studies using whole-genome/exome sequencing (WGS/WES) and transcriptome sequencing, and patient-reported outcomes. The sample size and power calculations were based on Simon’s optimal two-stage design. Poor and good response rates were defined as 10% and 25%, respectively (alpha error: 5%; beta error: 20%), resulting in a first stage of 18 patients and, if three or more patients responded continuation of the study with a target of a total of 43 patients. In the final analysis it was planned to reject the null hypothesis if eight or more responses were observed in 43 patients. The classification of response rates as poor or good reflects whether patients achieved DCR following six cycles of treatment. As of October 2020, 3 out of 10 enrolled patients achieved a DCR after six cycles allowing the continuation of the study. Recruitment was stopped due to slow accrual on 30 April 2022 when 7 out of 28 patients showed SD at month 6 evaluation.

Patients’ characteristics were compared with the Wilcoxon rank sum test for continuous variables and Fisher’s exact test for categorical variables. The median follow-up time was computed using the reverse Kaplan–Meier estimate.^23^ The Kaplan–Meier method was used to estimate the distribution of PFS and OS.^24^ OS was calculated from the start of palbociclib treatment until death or censored at last follow-up. PFS was calculated from start of palbociclib treatment until progression or death, whichever occurred first, or censored at last follow-up. The confidence interval (CI) estimation for survival curves was based on the cumulative hazard function using the Greenwood formula for variance estimation. Log-rank tests were employed to compare survival curves between groups.

Analysis was carried out using SAS version 9.4.

### Assessment of responder phenotype by immunohistochemistry analysis

The molecular responder phenotype was determined on formalin-fixed paraffin-embedded or cryopreserved tumor tissue obtained not >12 months before study enrollment. Immunohistochemically, p16 loss and expression of CDK4/6 as well as RB and phosphorylated RB (pRB, site S780), was carried out as previously described (Supplementary Table S1, available at https://doi.org/10.1016/j.esmoop.2025.105498).^13^ Based on the staining patterns, a potential non-responder phenotype was defined as p16 positivity in >10% of the tumor and lacking expression of RB/pRBS780, CDK4, and CDK6. The potential responder phenotype was subdivided into three categories, depending on the extent of immunoreactivity of the cells: type 1 (pRB expressed in ≤30%), type 2 (pRB expressed in 30%-70%), and type 3 (pRB expressed in >70%) of the cells. Detection of molecular alterations in *CDKN2A, RB1, CDK4,* and *CDK6* followed an established clinical workflow within the DKFZ/NCT/DKTK MASTER Program (NCT05852522).^25^

### Molecular analysis

Genomic markers were assessed from WGS/WES and transcriptome analysis. Molecular results were discussed in a dedicated molecular tumor board at the NCT Heidelberg.^25^^,^^26^ For the oncoprint, all genomic alterations were filtered based on curated gene lists (Supplementary Table S2, available at https://doi.org/10.1016/j.esmoop.2025.105498)^10^^,^^27^ and further restricted to genes where variants occurred in at least two patients. Selected genes were also included despite lacking variant recurrence (Supplementary Table S2, available at https://doi.org/10.1016/j.esmoop.2025.105498). The oncoprint was created with the R package ComplexHeatmap (version 2.18.0). Supervised mutational signature analysis of somatic single-nucleotide variants was carried out using the R package YAPSA (version 1.28.0) on cosmic v2 signatures^28^ with optimized signature-specific cut-offs. Identification of significant recurrent copy number changes was done with GISTIC v2.0 with a confidence level of 0.9 and a standard q-value cut-off of 0.25.^29^

The PROGENy algorithm (R package progeny version 1.12.0) was employed to estimate the activity states of 14 tumorigenesis-related signaling pathways based on TPM (transcript per million) values obtained through RNA sequencing.^30^^,^^31^

### Data availability

Genomic and transcriptomic data have been deposited in the European Genome-Phenome Archive (https://www.ebi.ac.uk/ega/datasets) under accession EGAS00001007985.

## Results

### Trial population

Between January 2018 and April 2023, 42 patients were screened. Fourteen patients were considered ineligible for the study. Twenty-eight patients started treatment. Patients’ characteristics are summarized in Table 1. Of note, 52% of patients had progressive disease as the best response to the last systemic therapy.

**Table 1:** Patient characteristics

	Patients *n* = 28 (100%)
Sex, *n* (%)	
Female	7 (25)
Male	21 (75)
Age, years	
Median, range	59, 31-84
Performance status, *n* (%)	
ECOG PS 0	12 (43)
ECOG PS 1	14 (50)
ECOG PS 2	2 (7)
Primary tumor site, *n* (%)	
Sacrum	16 (58)
Mobile spine	8 (28)
Skull base	4 (14)
TNM classification (according to UICC), *n* (%)	
Stage I	2 (7)
Stage II	0
Stage III	2 (7)
Stage IV	19 (68)
Not available	5 (18)
Histology^a^	
Conventional	27 (96)
Dedifferentiated	1 (4)
Years from diagnosis, median	
Median, range	7.7, 0-28
Number of previous systemic treatment lines	
Median, range	1, 0-5
Previous treatment, *n* (%)	
Surgery	27 (96)
Radiation	27 (96)
Systemic treatment	21 (75)
Imatinib	16
Erlotinib (± bevacizumab)	3
Sirolimus (± imatinib)	5
Sorafenib	3
Sunitinib	1
Doxorubicin/ifosfamide	1
Pembrolizumab	1
Best response to last line of systemic treatment	***n* = 21 (100%)**
Partial response, *n* (%)	1 (5)
Stable disease, *n* (%)	4 (24)
Progressive disease, *n* (%)	11 (52)
Missing, *n* (%)	5 (14)

ECOG, Eastern Cooperative Oncology Group; PS, performance status; TNM, tumor–node–metastasis; UICC, Union for International Cancer Control.

a According to reference pathology.

### Efficacy

**Figure 1:**
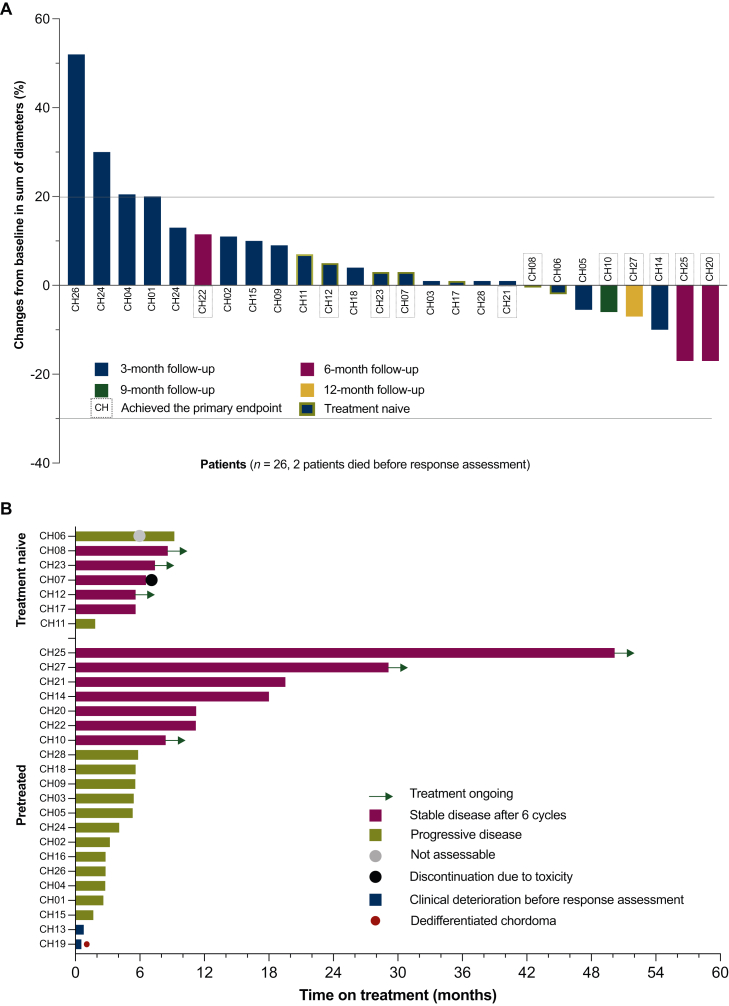

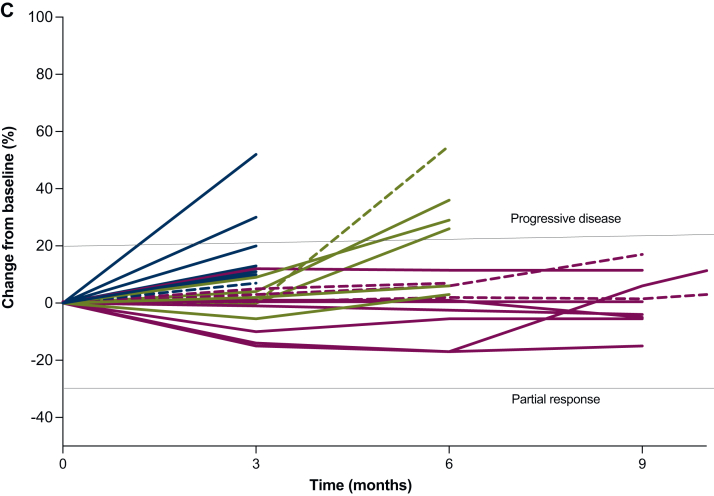
**Treatment outcomes.** (A) Waterfall plot showing maximum reduction in the sum of longest diameters of target tumor lesions per RECISTv1.1. Two patients who died before the first imaging assessment are not shown. Best response achieved during the trial was stable disease. (B) Swimmer plot showing duration on treatment for each patient. One patient was not assessable for RECIST at the 6-month follow-up due to palliative radiotherapy of one targeted lesion but still continued treatment. One patient discontinued treatment due to persistent neutropenia grade 3. Until the data cut-off, six patients were still on treatment. (C) Spiderplot showing RECIST pattern of response. Three patterns of response were observed: disease stabilization over several months (purple lines, *n* = 11 patients), initial stable disease followed by progression (green lines, *n* = 7 patients), and rapid progressing disease within the first 3 months, including non-target lesions (blue lines, *n* = 7 patients). Dashed lines represent therapy-naive patients. Two patients had no FU assessment and are not shown here. One patient not assessable for RECIST at FU2 was also excluded from this plot. FU1, follow-up 1; FU2, follow-up 2.

**Figure 2:**
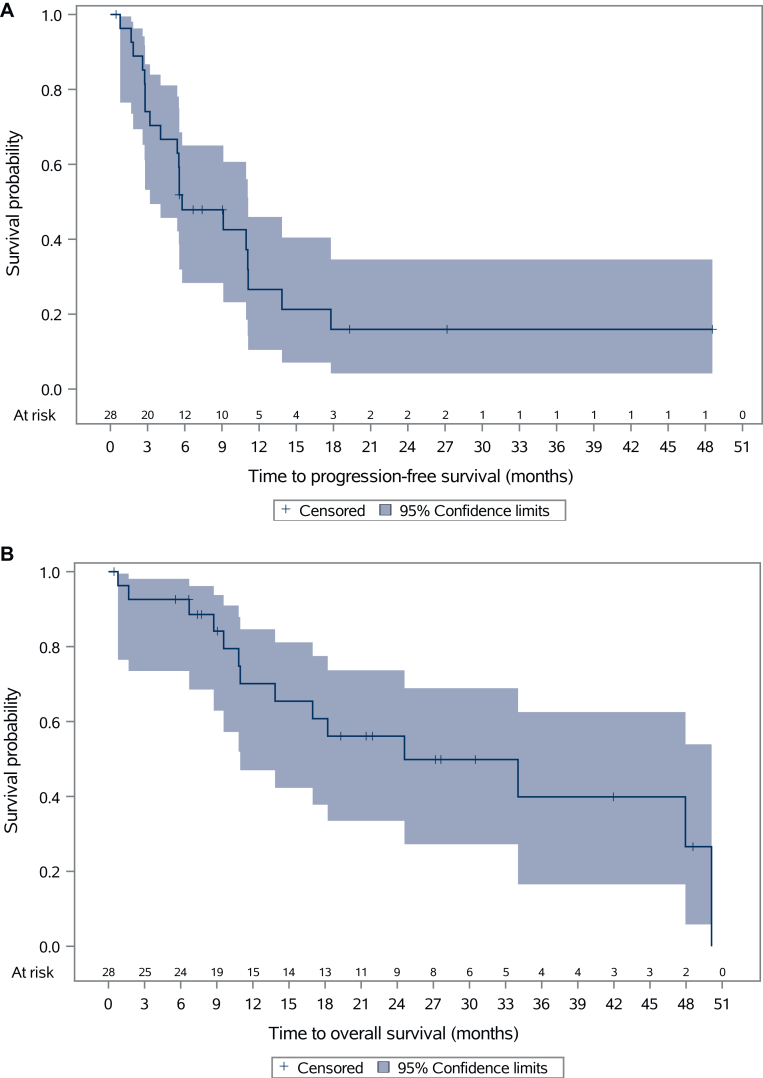
**Survival analysis**. (A) Progression-free survival in the safety population: 5.6 months (95% CI 3.2-11.1 months). (B) Overall survival in the safety population**:** 24.6 months (95% CI 11 months-not reached).CI, confidence interval.

At the data cut-off in December 2022, the median follow-up was 28.0 months (95% CI 20.0-48.6 months). The DCR after six cycles was achieved in 11 out of 28 patients (39%; 95% CI 21.5% to 59.4%). No PR or CR were obtained (Figure 1A, Supplementary Table S3, available at https://doi.org/10.1016/j.esmoop.2025.105498). Until the data cut-off, 6 patients (21%) were still on treatment and 22 (79%) discontinued treatment. Progressive disease was the cause of treatment discontinuation in 18 patients. One patient discontinued treatment due to toxicity. Two patients could not undergo response evaluation due to clinical deterioration shortly after treatment initiation, and one patient discontinued treatment per investigators decision due to clinical deterioration (Figure 1B). As depicted in the spider plot (Figure 1C, Supplementary Table S3, available at https://doi.org/10.1016/j.esmoop.2025.105498), the best tumor response within the range of SD mostly occurred within the first 3 months of treatment. An improvement in overall health status, with decreased pain medication intake and better mobility, was observed in some patients during follow-up study visits in this period by investigators. As per RECISTv1.1, most patients were assigned to one of the following patterns: disease stabilization over several months (*n* = 11), SD for the first 3 months followed by progress (*n* = 7), and rapid progress within the first 3 months (*n* = 7). Three patients achieved longer-term disease stabilization over 19, 27, and 42 months, respectively. All three patients had imatinib as the last therapy line before study enrollment, with SD as best response in one patient and PD in the other two (Figure 1C, Supplementary Table S4, available at https://doi.org/10.1016/j.esmoop.2025.105498). The median PFS among 28 assessable patients was 5.6 months (95% CI 3.2-11.1 months), and the median OS was 24.6 months (95% CI 11 months-not reached) (Figure 2A and B). The median PFS and median OS between pretreated and treatment-naive patients, accepted for enrollment only after the amendment, showed no significant difference with a median PFS of 5.5 months versus 9.1 months (*P* = 0.57) and median OS of 34.0 months versus 17.0 months (*P* = 0.91) (Supplementary Figure S1A and B, available at https://doi.org/10.1016/j.esmoop.2025.105498). Further clinical parameters had no significant impact on PFS and OS (Supplementary Table S5, available at https://doi.org/10.1016/j.esmoop.2025.105498).

In addition to RECISTv1.1 criteria, 16 patients were centrally reviewed by the Choi criteria.^32^ In 13 cases, Choi-based response assessment was not applicable (use of magnetic resonance imaging or suboptimal contrast enhancement). For the three assessable patients, we considered baseline and first follow-up imaging at 3 months as comparison time points. This time point corresponded to the most profound tumor reduction across all cases. One patient with clival chordoma with SD on RECIST had tumor attenuation with a 15% decrease in Hounsfield units (HU), consistent with responder phenotype according to the Choi criteria (Supplementary Figure S2, available at https://doi.org/10.1016/j.esmoop.2025.105498). However, the patient progressed at the second follow-up after six cycles. The other two patients with SD at 3 months follow-up, per RECISTv1.1, had a tumor attenuation of <15% HU.

### Toxicity

Treatment-related adverse events (TRAEs) occurred in 27 (96.4%) patients. Most TRAEs were limited to grade 1 or 2. Grade 3 TRAEs occurred in 11 (39.2%) patients, with the most frequent event being neutropenia in 8 (28.5%) patients. Five patients experienced infectious complications with grade 3 reported in one patient and neutropenic sepsis with a fatal outcome in one patient. Other hematological events affecting >10% of patients were limited to grades 1 and 2. Non-hematological events encountered in at least 10% of the patients were dry skin, headache, nausea, and fatigue, the majority of which were grades 1 and 2. Temporary treatment interruptions or dose reductions due to TRAEs were necessary in eight patients. Definitive treatment discontinuation was required in one patient due to neutropenia grade 3 (Table 2 and Supplementary Table S3, available at https://doi.org/10.1016/j.esmoop.2025.105498).

**Table 2:** Treatment-related adverse events occurring in ≥10% of patients

	Any grade *n* (%)	Grade 1-2 *n*	Grade 3 *n*	Grade 4 *n*	Grade 5 *n*
Anemia	6 (21.4)	6	0	0	0
Leukopenia	11 (39.3)	9	2	0	0
Neutropenia	16 (57.1)	7	8	0	1^a^
Thrombocytopenia	6 (21.4)	6	0	0	0
Infections	5 (17.9)	3	1	0	1^a^
Nausea	5 (17.9)	5	0	0	0
Fatigue	6 (21.4)	5	1	0	0
Headache	3 (10.7)	3	0	0	0
Dry skin	3 (10.7)	3	0	0	0

a One patient experienced febrile neutropenia with fatal outcome.

### Biomarker analysis

#### Distribution of responder phenotypes

IHC-based stratification in potential responder or non-responder phenotypes was part of the initial screening for all patients. All samples were negative for p16; 20 samples (71%) were positive for CDK4, 21 (75%) for CDK6, and 28 (100%) for RB/pRBS789. One patient tissue sample was negative for both CDK4 and CDK6; however, mRNA expression for *CDK4* and *CDK6* was not decreased and the patients were deemed eligible. CCND1 staining was available in 17 cases, of which 8 (47%) were positive. Ki67 index varied between 1% and 50% with a median value of 5% (Supplementary Table S1, available at https://doi.org/10.1016/j.esmoop.2025.105498). Based on baseline IHC stratification, 27 (96%) of the patients were categorized as potential responder phenotypes. Among them, 20 (71%) were identified as best assumed responder phenotype type 3, 7 (25%) as type 2, and 1 patient was classified as type 0, due to negativity for both CDK4 and CDK6 proteins. Next, we thought of investigating whether the immunohistochemical scores for CDK4, CDK6, and RB/pRB780S impact PFS and OS. There was no significant difference between the categories CDK4^IHC 0-1+^ versus CDK4^IHC 2-3+^, CDK6^IHC 2-3+^ versus CDK6^IHC 0-2+^, or RB/pRB780S^IHC 3+^ versus RB/pRB780S^IHC 0-2+^ in terms of median PFS or median OS. When considering the CDK4/CDK6^IHC 2-3+^ group the difference was again not significant (Supplementary Table S5, available at https://doi.org/10.1016/j.esmoop.2025.105498).

#### Landscape of genomic alterations

**Figure 3:**
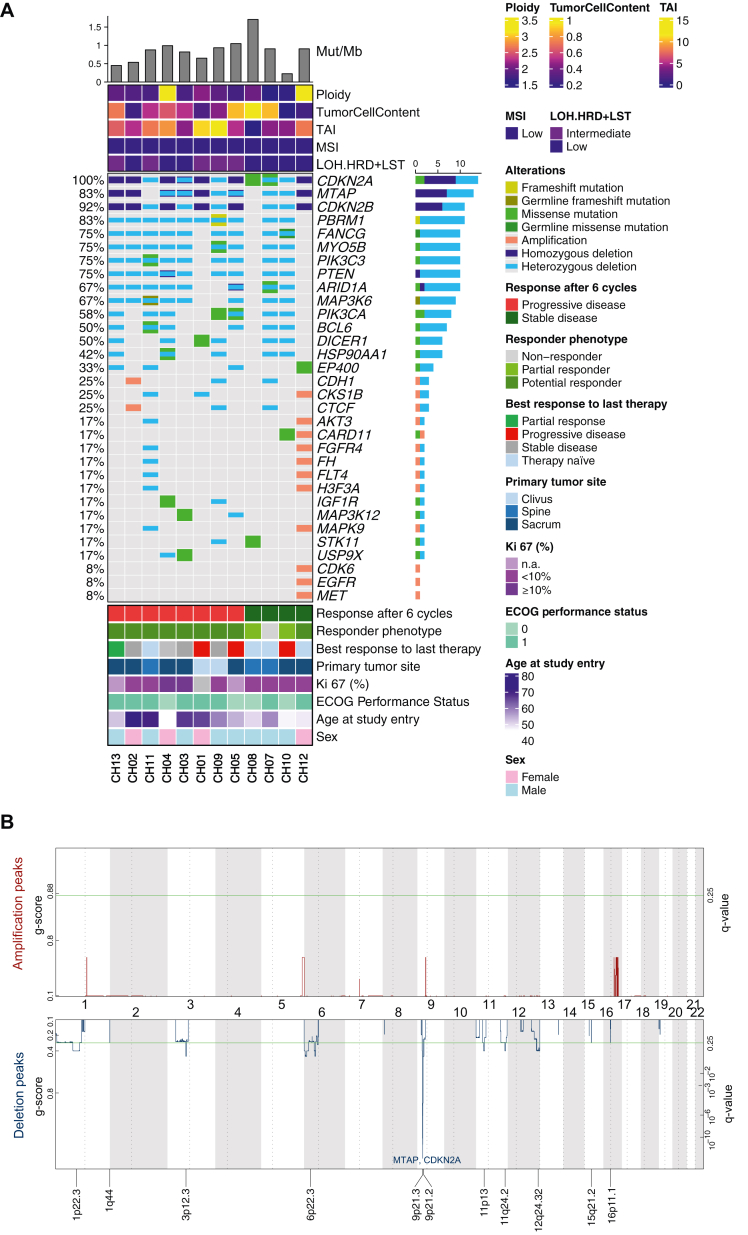
**Genomic landscape and copy number plot.** (A) Oncoprint with the genomic alterations and clinical features of 12 patients with genomic data. (B) Copy number profile showing 9p21 as the most significant deleted region across the 12 analyzed samples.

Twelve samples collected before study treatment could be evaluated for WGS (*n* = 10) and WES (*n* = 2). Transcriptomic analysis (RNA-seq) was possible for seven samples. Genomic alterations are illustrated in the oncoprint in Figure 3A. Tumor mutation burden was low, with a median of 0.8 mutations/Mb (range 0.2-1.7 mutations/Mb), and all analyzed samples were microsatellite stable. *CDKN2A* alterations affected all cases, with homozygous deletions detected in eight patients and heterozygous losses in four cases. In addition, two patients harbored inactivating mutations in *CDKN2A* (H98P and S12X). The size of the *CDKN2A* loss varied between 115kB and1060kB. In seven out of eight cases with *CDKN2A* homozygous deletions, homozygous *MTAP* losses co-occurred. *MTAP* is located on 9p21.3, ∼22kb telomeric to CDKN2A, is often co-deleted with CDKN2A, and encodes for the enzyme methylthioadenosine phosphorylase, a regulator of polyamine metabolism.^33^^,^^34^ Mutations in the Pi3K/mTOR pathway occurred in four cases with *PIK3CA* oncogenic mutations (E542K, E545K, K100R) in three patients and a *PTEN* homozygous deletion in one patient, respectively. These findings enabled later treatment with the PIK3CA inhibitor inavolisib (NCT04551521). Mutations affecting chromatin-modeling genes were identified in three cases (*ARID1A, n* = 2 and *PBRM1, n* = 1). Further, *EGFR, MET, and CDK6* amplification were detected in one sample. The merged copy number profile of the analyzed samples indicated a peak deletion affecting the 9p21 region (Figure 3B). Mutational signatures were enriched for the AC3 signature, associated with defective homologous recombination-based DNA double-strand break repair and the APOBEC signatures AC2 and AC13 as previously reported^11^ (Supplementary Figure S3, available at https://doi.org/10.1016/j.esmoop.2025.105498).

#### Correlation between baseline IHC expression, baseline mRNA expression, and CDKN2A copy number

Pretreatment mRNA expression levels for *CDK4* and *CDK6* were compared with tissue expression determined by IHC. There was no significant association between the IHC 3+, IHC 2+, IHC 1+, or negative score groups and the mRNA expression of CDK4 and CDK6. The correlation test for CCND1 between mRNA expression and IHC score was omitted due to the absence of IHC results in three out of seven cases. For RB1, the score also includes pRBS780 expression, a post-translational modification not reflected in mRNA. Expression of p16 based on IHC was negative in all samples. Interestingly, mRNA expression levels differed between cases with homozygous and heterozygous *CDKN2A* deletion. mRNA expression levels were significantly lower in cases with homozygous deletions than in cases with heterozygous deletions (*P* = 0.034, Wilcoxon rank test). However, given the limited sample size for available RNA-seq, these results must be cautiously interpreted and need further validation (Supplementary Table S7, available at https://doi.org/10.1016/j.esmoop.2025.105498).

#### RNA-based pathway activity inference

To identify deregulated signaling pathways and understand potential reasons for treatment resistance, we employed the PROGENy algorithm (Supplementary Figure S4, available at https://doi.org/10.1016/j.esmoop.2025.105498). Despite the small sample size, the findings suggest the presence of different deregulated pathways at study entry that can impact response and promote resistance during treatment. In patient CH08, Pi3K signaling was increased, consistent with the underlying oncogenic *PIK3CA* mutation. Unfortunately, for the other two patients with oncogenic *PIK3CA* mutations, RNA-seq data were not available. Further, transcriptomic analysis revealed deregulated pathways not captured by genomic data. For instance, in patient CH03, the vascular endothelial growth factor and hypoxia pathways were predominantly enriched, suggesting angiogenesis as a driving mechanism of tumor proliferation and providing a rationale for antiangiogenic therapy. While no specific oncogenic driver was identified, WGS copy number analysis indicated heterozygous losses of chromosomes 3p and 10q, where the *VHL* and *PTEN* genes are located (Supplementary Figure S5, available at https://doi.org/10.1016/j.esmoop.2025.105498). Both genes are associated with increased hypoxia response.^30^ Similarly, a strong p53/DDR pathway activity was observed in patient CH01 lacking a genomic correlate. This patient experienced progress within the first 3 months of treatment. An interesting finding in both cases was the low expression of the estrogen pathway compared with the other patients’ samples.

## Discussion

In this phase II trial, we evaluated treatment with palbociclib in advanced chordomas using genomic markers for treatment stratification. Palbociclib monotherapy met the prespecified endpoint with a DCR of 39%, with SD as the best response. Three patients derived a clinical benefit over 18 months, and although no objective responses were recorded, tumor regression observed in some cases suggests that palbociclib possesses antitumor activity. The median PFS and median OS of the study cohort were 5.6 months and 24.6 months, respectively. Of note, the median OS was comparatively long, reflecting both the slow growth nature of the disease and that some patients can further benefit from subsequent treatments. We could not identify any significant survival benefit in treatment-naive patients compared with pretreated patients, despite the 3.6-month difference in median PFS in favor of therapy-naive patients. The results must, however, be interpreted with caution due to the small number of patients in the therapy-naive versus pretreated group. Notably, the median OS was longer for the pretreated group, which likely reflects the treatment heterogeneity rather than a specific biological behavior. Differences in surgical and radiotherapy strategies across institutions, with some patients receiving systemic therapy earlier in the disease course and others benefitting from local modalities, further contribute to this heterogeneity.

The results of our trial align with previous phase II trials on targeted treatments in chordoma in terms of response; however, median PFS is shorter.^6^^,^^8^^,^^35^^,^^36^ In the phase II trial with imatinib in PDGFRB/PDGFB-expressing chordomas, the clinical benefit rate (CBR) (CR + PR + SD per RECIST) was 64% after 6 months, with 1 PR and 30 SDs and a median PFS of 9 months. The differences in CBR and median PFS could be attributed to the heterogeneous patient population and differences in line of treatment. In our study, up to 75% of the patients enrolled had pretreated advanced or metastatic disease at study entry. Among them, 52% had PD as their best response to the last systemic treatment, including 15 patients (71%) who progressed on imatinib. Comparable results were reported also for lapatinib, a dual EGFR and human epidermal growth factor 2 (HER2) inhibitor, investigated in a phase II trial in patients with EGFR-expressing chordoma. Response assessment per RECISTv1.1 yielded no objective response with a CBR after 6 months of 22% and a median PFS of 8 months. Most patients in this trial were imatinib pretreated as well. Encouraging PFS results were reported for sorafenib, a kinase inhibitor targeting some of the receptor tyrosine kinases known to be expressed in chordomas (PDGFRB and VEGFR1-3).^37^ After a short median follow-up time of 8.7 months, the median OS and median PFS were not reached, and the 6-month progression-free rate was 85.3%. Yet, only one patient achieved a PR per RECISTv1.1. Patient enrollment was not biomarker-driven and 44% of patients were treatment-naive. Thus, a common observation emerging from our study and the trials discussed above is the low rate of objective responses achieved with single-agent targeted therapies, despite differences in median PFS. This suggests that currently available drugs or their use as monotherapy might not be sufficient to achieve deeper tumor responses. Regarding response assessment, it is also worth mentioning that the imaging methods employed varied between studies. Previous trials have shown that the Choi criteria might be more effective in capturing the biological effects of targeted therapies, particularly in slow-growing tumors like chordoma.^3^ However, due to the very limited number of patients assessed by the Choi criteria, we could reproduce this finding in only one patient whose response was upgraded to PR after using the Choi criteria. These observations further underscore the importance of longitudinal tumor assessment, which may help identify patterns of response otherwise missed by RECISTv1.1 and facilitate earlier treatment intervention. Other imaging techniques, such as positron emission tomography (PET)-computed tomography scan using [^18^F]2-fluoro-2-deoxy-D-glucose, showed a decrease in PET uptake with results comparable to PR per Choi.^8^^,^^35^^,^^38^ Given the differences observed across imaging techniques and their potential therapeutic implication, novel imaging methods should be considered for upcoming clinical trials.

The toxicity profile was consistent with previous trial results,^20^^,^^21^^,^^39^^,^^40^ with neutropenia being the main cause of grade 3 or higher adverse events encountered in 39% of patients. Still, toxicity was manageable with dose adjustment and temporary interruption, with one permanent treatment discontinuation due to neutropenia, making palbociclib a good candidate for combination therapies.

The protein profiles established in chordoma cell lines and the tissue immunoreactivity of various cell cycle components stratify the majority of chordoma patients as potential responders to palbociclib treatment, and the degree of pRBS780 phosphorylation has been identified as the most powerful predictor of treatment response.^13^ However, in our trial, this phenotype was not confirmed as a predictor of response. Moreover, seven patients with the type 3 phenotype experienced earlier disease progression within the first 3 months of treatment. Regarding expression of other genes included in the IHC panel, we could not detect any correlation between the IHC score and mRNA expression levels. In a recent phase II study with palbociclib conducted in patients with different sarcoma types stratified based on the overexpression of *CDK4* without *CDKN2A* overexpression using quantitative RT–PCR, the authors identified higher immunohistochemical scores of CDK4 protein expression associated with better survival.^22^ Moreover, *CDK4* mRNA expression levels highly correlated with the immunohistochemical scores. Due to limited available RNA sequencing data, we cannot draw any conclusions regarding the predictive value of *CDK4* mRNA expression for this chordoma cohort. In our dataset, higher CDK4 or CDK6 IHC scores did not predict for a PFS or OS advantage. However, compared with the study by Martin-Broto et al.,^22^ the immunohistochemical scoring used in our trial did not differentiate between the strength and extension of protein expression, considering only the extension of the expression. Therefore, this approach questions whether the various degrees of protein expressions are reproduced reliably as compared with quantitative methods such as mRNA expression.

Exploratory genomic analysis using WGS/WES identified various *CDKN2A* alterations with homozygous deletions as the most common alteration and 9p21 as the most significant deleted region. This is a critical genomic region associated with cell cycle regulation, tumor suppression, and immune response. Genes located in that region comprise *CDKN2A, CDKN2B, MTAP*, and interferon-α genes, among others. Co-deletion of *CDKN2A* and *MTAP* due to its proximity occurs frequently across cancers.^34^^,^^41^ This finding is of therapeutic relevance as *MTAP-*deficient cells are highly dependent on protein arginine methyltransferase 5 (PRMT5)*,* an arginine methyltransferase, conferring vulnerability to PRMT5 inhibition. PRMT5 inhibitors are currently under investigation in early-phase clinical trials with promising results across different advanced solid tumors.^42^ Importantly, this co-deletion is captured only by genomic analysis using WGS or WES or using IHC assays. Furthermore, genomic analysis identified additional potential targets for molecularly tailored therapies, including *MET* amplifications or *PIK3CA* mutations.^43^

Primary resistance mechanisms to CDK4/6 inhibitors can emerge through parallel signaling activated upstream of the cell cycle pathway. Oncogenic mutations that cause PI3K/AKT/mTOR pathway activation drive resistance to CDK4/6 inhibitors in hormone receptor (HR)-positive human epidermal growth factor receptor 2 (HER2)-negative metastatic breast cancer.^44^^,^^45^ In our study, four patients harbored mutations in *PIK3CA* and *PTEN,* three of them enriched in patients with early progress on palbociclib. In addition to the PI3K/AKT/mTOR pathway deregulation, other transcriptomic features such as reduced estrogen pathway activity or increased p53 activity have been recently associated with decreased response to CDK4/6 inhibition in HR-positive HER2-negative metastatic breast cancer.^44^^,^^45^ These findings need further validation in larger chordoma cohorts and support combination therapies as the more effective treatment approach. The results of our study compare favorably with previous studies that identified PDGFRB/PDGF, PI3K/AKT, RAS/MAPK, or EGFR as recurrently dysregulated pathways in chordomas.^37^ The combination of mTOR inhibitors with imatinib was investigated in case series and a phase II trial with encouraging results. However, treatment-related toxicity may pose a significant concern for combination treatments, as high rates of permanent toxicity-induced treatment discontinuation have been observed.^9^^,^^38^

## Conclusions

In summary, this trial of a molecularly guided chordoma therapy reached its primary endpoint, demonstrating a modest antitumor effect, though signals of activity were observed. No specific clinical or molecular predictors of response were identified. However, combined genomic and transcriptomic profiling can identify additional therapeutic targets and potential resistance mechanisms to CDK4/6 inhibitors. The molecular heterogeneity of the disease suggests that combination therapies could be more effective but there is clearly a continuing need for more, molecular mechanism-aware therapies or combination strategies, as well as better patient selection. We encourage considering genomic analysis for all chordoma patients who are candidates for systemic therapies. Additionally, having more active centers would have improved recruitment and expanded study access. We hope this last challenge can be overcome in the newly established network of NCTs in Germany, including 11 comprehensive cancer centers that have defined rare cancers as one of their priority disease areas.

## Supplementary data

Supplementary Table 3

Supplemenatry Table 4
