# Supplementary material for: CDK4/6 inhibition in advanced chordoma: final results of the NCT PMO-1601 trial
Source: ESMO Open. 2025 Jul 7;10(7):105498. doi: 10.1016/j.esmoop.2025.105498 (PMC12272896; doi:10.1016/j.esmoop.2025.105498)
Supplement: Supplementary Table 1 [file mmc1.docx]

**Table S1. Responder phenotypes by Immunohistochemistry**

| **ID** | **Ki67** | **p16** | **CDK4** | **CDK6** | **CCND1** | **Rb/pRBS780** | **INI1** | **Histology Type** | **Responder-Phenotype** | **6-Months**  **Response** |
| --- | --- | --- | --- | --- | --- | --- | --- | --- | --- | --- |
| CH01 | n. a | 0 | 1 | 3 | n.a | 3 | positive | 9370/3 Conventional chordoma | potential RP | PD |
| CH02 | 10% | 0 | 0 | 3 | n.a | 3 | positive | 9370/3 Conventional chordoma | potential RP | PD |
| CH03 | 5-10% | 0 | 0 | 0 | n.a | 1 | positive | 9370/3 Conventional chordoma | non-responder | PD |
| CH04 | 10-15% | 0 | 1 | 3 | 0 | 3 | n.d. | 9370/3 Conventional chordoma | potential RP | PD |
| CH05 | 1% | 0 | 3 | 3 | 3 | 2 | n.d. | 9370/3 Conventional chordoma | potential RP | PD |
| CH06 | 5% | 0 | 3 | 0 | 0 | 3 | positive | 9370/3 Conventional chordoma | potential RP | PD |
| CH07 | 50% | 0 | 1 | 1 | 0 | 3 | positive | 9370/3 Conventional chordoma | potential RP | SD |
| CH08 | 5% | 0 | 3 | 1 | 0 | 3 | positive | 9370/3 Conventional chordoma | potential RP | SD |
| CH09 | 5% | 0 | 1 | 1 | 0 | 3 | positive | 9370/3 Conventional chordoma | potential RP | PD |
| CH10 | 10% | 0 | 1 | 1 | 3 | 2 | positive | 9370/3 Conventional chordoma | potential RP | SD |
| CH11 | 5% | 0 | 3 | 3 | 3 | 2 | n.d. | 9370/3 Conventional chordoma | potential RP | PD |
| CH12 | 5% | 0 | 1 | 3 | 2 | 3 | positive | 9370/3 Conventional chordoma | potential RP | SD |
| CH13 | 1-5% | 0 | 0 | 1 | n.a | 3 | positive | 9370/3 Conventional chordoma | potential RP | PD |
| CH14 | 10-20% | 0 | 1 | 2 | n.a | 3 | positive | 9370/3 Conventional chordoma | potential RP | SD |
| CH15 | 5% | 0 | 1 | 3 | n.a | 3 | positive | 9370/3 Conventional chordoma | potential RP | PD |
| CH16 | 5% | 0 | 3 | 1 | n.a | 3 | positive | 9370/3 Conventional chordoma | potential RP | PD |
| CH17 | 5% | 0 | 2 | 0 | 3 | 3 | positive | 9370/3 Conventional chordoma | potential RP | PD |
| CH18 | 5% | 0 | 0 | 1 | 0 | 3 | positive | 9370/3 Conventional chordoma | potential RP | PD |
| CH19 | 5% | 0 | 0 | 1 | 0 | 3 | negative | 9372/3 Dedifferentiated chordoma | potential RP | PD |
| CH20 | 5% | 0 | 2 | 3 | 3 | 2 | positive | 9370/3 Conventional chordoma | potential RP | SD |
| CH21 | 1% | 0 | 2 | 0 | 3 | 3 | positive | 9370/3 Conventional chordoma | potential RP | SD |
| CH22 | 5% | 0 | 2 | 3 | 0 | 2 | n.d. | 9370/3 Conventional chordoma | potential RP | SD |
| CH23 | 5% | 0 | 1 | 2 | 3 | 3 | positive | 9370/3 Conventional chordoma | potential RP | SD |
| CH24 | 5% | 0 | 0 | 1 | n.a | 3 | positive | 9370/3 Conventional chordoma | potential RP | PD |
| CH25 | 1% | 0 | 0 | 3 | n.a | 3 | positive | 9370/3 Conventional chordoma | potential RP | SD |
| CH26 | 5% | 0 | 0 | 1 | n.a | 3 | positive | 9370/3 Conventional chordoma | potential RP | PD |
| CH27 | 5% | 0 | 2 | 0 | 3 | 2 | positive | 9370/3 Conventional chordoma | potential RP | SD |
| CH28 | n. a | 0 | 3 | 0 | n.a | 2 | positive | 9370/3 Conventional chordoma | potential RP | PD |

**Legend:**

0 negative staining

1 ≤30% of the cells positive

2 30-70% of cells positive

3 >70% of cells positive

p16 negative ≤10% of cells are positive

**Non-responder phenotype:**

p16 positivity in over 10% of the tumor and lacking expression of RB, pRB, CDK4, and CDK6

**Potential responder phenotype**:

type 1= pRB expressed in ≤30% of the cells (+)

type 2= pRB expressed in 30-70% of the cells (++)

type 3= pRB expressed in >70% of the cells (+++)

SD - stable disease

PD - progressive disease

n.a. not available

**Antibodies used for immune staining**

**Ki-67**  clone MIB-1; Dianova, 1:200, M7240

**p16**  Santa Cruz Biotechnology, 1:100, Sc-56330

**CDK4** Zytomed Systems, 1:100, 603-1840

**CDK6** Abcam, 1:500, Ab 54576

**Cyclin D1** SP4, DCS, Cl677C01

**Rb**  Cell Signaling Technology, 1:100, 9309

**pRb** Ser780; Cell Signaling Technology, 1:100, 9307
