# Supplementary material for: CDK4/6 inhibition in advanced chordoma: final results of the NCT PMO-1601 trial
Source: ESMO Open. 2025 Jul 7;10(7):105498. doi: 10.1016/j.esmoop.2025.105498 (PMC12272896; doi:10.1016/j.esmoop.2025.105498)
Supplement: Supplementary Table 3 [file mmc3.docx]

**Table S3. RECIST v. 1.1 Results**

| **ID** | **Current Cycle** | **Sum of Measurement (mm)** | **∆ %** | **Target** | **Target lesions response (Overall Response)** | **Comment** |
| --- | --- | --- | --- | --- | --- | --- |
| **CH01** | Baseline (V0) | 106 |  | Target | . |  |
|  | Cycle 4 W1 D1(V8) | 127 | +20 | Target | PD |  |
| **CH02** | Baseline (V0) | 132 |  | Target | . |  |
|  | Cycle 4 W1 D1(V8) | 147 | +11 | Target | SD | Progress at non-target site |
| **CH03** | Baseline (V0) | 64 |  | Target | . |  |
|  | Cycle 4 W1 D1(V8) | 64 | 0 | Target | SD |  |
|  | Cycle 5 W1 D1 (V9) | 65 | +2 | Target | SD |  |
|  | Cycle 6 D29 (V11) | 68 | +6 | Target | PD | Progress at non-target site |
| **CH04** | Baseline (V0) | 73 |  | Target | . |  |
|  | Cycle 4 W1 D1(V8) | 88 | +20.5 | Target | PD |  |
| **CH05** | Baseline (V0) | 331 |  | Target | . |  |
|  | Cycle 4 W1 D1(V8) | 313 | -5.5 | Target | SD | Clinical progress after cycle 5 with no radiologic assessment after cycle 6 |
| **CH06** | Baseline (V0) | 184 |  | Target | . |  |
|  | Cycle 4 W1 D1(V8) | 180 | -2 | Target | SD |  |
|  | Cycle 6 D29 (V11) | 20 | x | Target | Not evaluable per RECIST | Palliative RT of target lesion |
|  | Cycle 10 D1 (V14) | 23 |  | Target | PD |  |
| **CH07** | Baseline (V0) | 130 |  | Target | . |  |
|  | Cycle 4 W1 D1(V8) | 134 | +3 | Target | SD |  |
|  | Cycle 6 D29 (V11) | 138 | +6 | Target | SD |  |
| **CH08** | Baseline (V0) | 94 |  | Target | . |  |
|  | Cycle 4 W1 D1(V8) | 64 | 1 | Target | SD |  |
|  | Cycle 6 D29 (V11) | 76 | 2 | Target | SD |  |
|  | Cycle 8 D 1 (V12) | 76 | 2 | Target | SD |  |
| **CH09** | Baseline (V0) | 45 |  | Target | . |  |
|  | Cycle 4 W1 D1(V8) | 49 | +9 | Target | SD |  |
|  | Cycle 7 D1 (V11a) | 58 | +29 | Target | PD |  |
| **CH10** | Baseline (V0) | 117 |  | Target | . |  |
|  | Cycle 4 W1 D1(V8) | 116 | -1 | Target | SD |  |
|  | Cycle 6 D29 (V11) | 114 | -3 | Target | SD |  |
|  | Cycle 10 D1 (V14) | 112 | -4 | Target | SD |  |
| **CH11** | Baseline (V0) | 72 |  | Target | . |  |
|  | Cycle 4 W1 D1(V8) | 77 | +7 | Target | SD | Progress at non-target lesion |
| **CH12** | Baseline (V0) | 84 |  | Target | . |  |
|  | Cycle 4 W1 D1(V8) | 88 | +5 | Target | SD |  |
|  | Cycle 6 D29 (V11) | 90 | +7 | Target | SD | Lost to FU |
| **CH13** | Baseline (V0) | 145 |  | Target | . | Clinical deterioration, no FU assessment |
| **CH14** | Baseline (V0) | 92 |  | Target | . |  |
|  | Cycle 4 W1 D1(V8) | 82 | -10 | Target | SD |  |
|  | Cycle 6 D29 (V11) | 87 | -5.5 | Target | SD |  |
|  | Cycle 10 D1 (V14) | 87 | -5.5 | Target | SD |  |
|  | Cycle 13 D1 (V17) | 10 | x | Target | SD |  |
|  | Cycle 16 D1 (V20) | 10 | x | Target | SD |  |
|  | Cycle 19 D1 (V23) | 11 | x | Target | PD |  |
| **CH15** | Baseline (V0) | 178 |  | Target | . |  |
|  | Cycle 6 D29 (V11) | 196 | +10 | Target | PD | Progress before FU1. Imaging taken before FU1 |
| **CH16** | Baseline (V0) | 122 |  | Target | . |  |
|  | Cycle 4 W1 D1(V8) | 159 | +30 | Target | PD |  |
| **CH17** | Baseline (V0) | 49 |  | Target | . |  |
|  | Cycle 4 W1 D1(V8) | 49 | 0 | Target | SD |  |
|  | Cycle 6 D29 (V11) | 76 | +55 | Target | PD |  |
| **CH18** | Baseline (V0) | 105 |  | Target | . |  |
|  | Cycle 4 W1 D1(V8) | 109 | +4 | Target | SD |  |
|  | Cycle 6 D29 (V11) | 143 | +36 | Target | PD |  |
| **CH19** | Baseline (V0) | 185 | x | Target | . | Clinical deterioration before FU1 |
| **CH20** | Baseline (V0) | 149 |  | Target | . |  |
|  | Cycle 4 W1 D1(V8) | 127 | -14 | Target | SD |  |
|  | Cycle 6 D29 (V11) | 123 | -17 | Target | SD |  |
|  | Cycle 10 D1 (V14) | 131 | +6 | Target | SD |  |
|  | Cycle 13 D1 (V17) | 149 | +22 | Target | PD |  |
| **CH21** | Baseline (V0) | 52 |  | Target | . |  |
|  | Cycle 4 W1 D1(V8) | 52 | 0 | Target | SD |  |
|  | Cycle 6 D29 (V11) | 52 | 0 | Target | SD |  |
|  | Cycle 9 D1 (V13) | 52 | 0 | Target | SD |  |
| **CH22** | Baseline (V0) | 166 |  | Target | . |  |
|  | Cycle 4 W1 D1(V8) | 187 | +12 | Target | SD |  |
|  | Cycle 6 D29 (V11) | 185 | +11.5 | Target | SD |  |
|  | Cycle 8 D 1 (V12) | 185 | +11.5 | Target | SD |  |
|  | Cycle 10 D1 (V14) | 185 | +11.5 | Target | SD |  |
|  | Cycle 13 D1 (V17) | 197 | 19 | Target | PD | Clinical Progress |
| **CH23** | Baseline (V0) | 124 |  | Target | . |  |
|  | Cycle 4 W1 D1(V8) | 128 | +3 | Target | SD |  |
|  | Cycle 6 D29 (V11) | 132 | +6 | Target | SD |  |
|  | Cycle 9 D1 (V13) | 146 | +17 | Target | SD |  |
| **CH24** | Baseline (V0) | 436 |  | Target | . |  |
|  | Cycle 5 W1 D1 (V9) | 492 | +13 | Target | PD | Clinical deterioration |
| **CH25** | Baseline (V0) | 47 |  | Target | . |  |
|  | Cycle 4 W1 D1(V8) | 41 | -15 | Target | SD |  |
|  | Cycle 6 D29 (V11) | 39 | -17 | Target | SD |  |
|  | Cycle 10 D1 (V14) | 42 | +8 | Target | SD |  |
|  | Cycle 13 D1 (V17) | 40 | +2.5 | Target | SD |  |
|  | Cycle 16 D1 (V20) | 42 | +8 | Target | SD |  |
|  | Cycle 19 D1 (V23) | 43 | +9 | Target | SD |  |
|  | Cycle 23 D1 (V27) | 41 | +5 | Target | SD |  |
|  | Cycle 26 D1 (V30) | 42 | +8 | Target | SD |  |
|  | Cycle 29 D1 (V33) | 43 | +10 | Target | SD |  |
|  | Cycle 32 D1 (V36) | 41 | +5 | Target | SD |  |
|  | Cycle 35 D1 (V39) | 42 | +8 | Target | SD |  |
|  | Cycle 39 D1 (V43) | 42 | +8 | Target | SD |  |
|  | Cycle 42 D1 (V46) | 40 | +5 | Target | SD |  |
|  | Cycle 45 D1 (V49) | 35 | -10 | Target | SD |  |
|  | Cycle 48 D1 (V52) | 35 | -10 | Target | SD |  |
|  | Cycle 50 D1 (V54) | 36 | -10 | Target | SD |  |
| **CH26** | Baseline (V0) | 104 |  | Target | . |  |
|  | Cycle 4 W1 D1(V8) | 158 | +52 | Target | PD |  |
| **CH27** | Baseline (V0) | 76 |  | Target | . |  |
|  | Cycle 4 W1 D1(V8) | 78 | +1 | Target | SD |  |
|  | Cycle 6 D29 (V11) | 77 | +1 | Target | SD |  |
|  | Cycle 11 D1 (V15) | 73 | -5 | Target | SD |  |
|  | Cycle 14 D1 (V18) | 71 | -7 | Target | SD |  |
|  | Cycle 17 D1 (V21) | 76 | +7 | Target | SD |  |
|  | Cycle 20 D1 (V24) | 76 | +7 | Target | SD |  |
|  | Cycle 23 D1 (V27) | 77 | +8 | Target | SD |  |
|  | Cycle 26 D1 (V30) | 81 | +14 | Target | SD |  |
|  | Cycle 29 D1 (V33) | 75 | +7 | Target | SD |  |
|  | Cycle 32 D1 (V36) | 76 | +7 | Target | SD |  |
| **CH28** | Baseline (V0) | 19 |  | Target | . |  |
|  | Cycle 4 W1 D1(V8) | 19 | 0 | Target | SD |  |
|  | Cycle 6 D29 (V11) | 24 | +26 | Target | PD |  |

**BL –** baseline, **FU**-follow-up, **SD –** stable disease**, PD-**progressive disease**, RT-**radiotherapy
