# Supplementary material for: CDK4/6 inhibition in advanced chordoma: final results of the NCT PMO-1601 trial
Source: ESMO Open. 2025 Jul 7;10(7):105498. doi: 10.1016/j.esmoop.2025.105498 (PMC12272896; doi:10.1016/j.esmoop.2025.105498)
Supplement: Supplemenatry Table 4 [file mmc4.docx]

**Table S4.** Outcome on last therapy prior to study entry

| **Patient ID** | **Last therapy prior to study entry** | **Best outcome** | **Reason for discontinuation and referral to study** | **Best response on palbociclib** | **Primary endpoint reached** |
| --- | --- | --- | --- | --- | --- |
| CH01 | Imatinib | SD | PD | PD | no |
| CH02 | Erlotinib/Bevacizumab | SD | PD | PD | no |
| CH03 | Doxorubicin/Ifosfamid | PD | PD | PD | no |
| CH04 | Imatinib | PD | PD | PD | no |
| CH05 | Imatinib | PD | PD |  | no |
| CH06 | **Treatment naïve**  Palliative radiotherapy sacral and of lumbar soft tissue manifestation | SD | Multifocal disease, progressive metastases | SD | no |
| CH07 | **Treatment naïve** | - | Local progress after surgery and proton radiotherapy | SD | **yes** |
| CH08 | **Treatment naïve** | - | Exhausted local therapy options; multifocal disease | SD | **yes** |
| CH09 | Pembrolizumab | SD | PD |  | no |
| CH10 | Imatinib | SD | PD | SD | **yes** |
| CH11 | **Treatment naïve** | - | Exhausted local therapy options; progressive soft tissue tumor at the resection margins thoraco-lumbar region | PD | no |
| CH12 | **Treatment naïve** | - | Progressive sacral chordoma after radiotherapy without surgery options | SD | **yes** |
| CH13 | Sorafenib | PR | PD | SD | no |
| CH14 | Imatinib | missing | PD | SD | **yes** |
| CH15 | Imatinib | missing | Missing | PD | no |
| CH16 | Imatinib | SD | PD | PD | no |
| CH17 | **Treatment naïve** | - | Skull base chordoma with local progress after surgery and proton radiotherapy | SD | no |
| CH18 | Imatinib | PD | PD | SD | no |
| CH19 | Sorafenib | missing | PD | PD | no |
| CH20 | Sirolimus | PD | PD | SD | **yes** |
| CH21 | Imatinib | Missing | Missing | SD | **yes** |
| CH22 | Imatinib | PD | PD | SD | **yes** |
| CH23 | **Treatment naïve** | - | Sacral chordoma with local progress after radiotherapy and local resections | SD | **yes** |
| CH24 | Erlotinib | PD | PD | PD | no |
| CH25 | Imatinib | PD | PD | SD | **yes** |
| CH26 | Imatinib | PD | PD | PD | no |
| CH27 | Imatinib | PD | PD | SD | yes |
| CH28 | Imatinib | SD | Missing | SD | no |

**SD** Stable Disease; **PD** Progressive Disease

| **Site ID** | **Study ID** | **Paper-ID** |
| --- | --- | --- |
| 01-001 | 2 | CH01 |
| 01-002 | 3 | CH02 |
| 01-007 | 25 | CH03 |
| 01-008 | 28 | CH04 |
| 01-009 | 31 | CH05 |
| 01-011 | 33 | CH06 |
| 01-013 | 35 | CH07 |
| 01-015 | 38 | CH08 |
| 01-016 | 39 | CH09 |
| 01-017 | 40 | CH10 |
| 01-018 | 42 | CH11 |
| 01-019 | 43 | CH12 |
| 02-001 | 1 | CH13 |
| 02-002 | 5 | CH14 |
| 02-003 | 6 | CH15 |
| 02-004 | 7 | CH16 |
| 02-006 | 11 | CH17 |
| 02-008 | 14 | CH18 |
| 02-009 | 15 | CH19 |
| 02-013 | 22 | CH20 |
| 02-014 | 26 | CH21 |
| 02-015 | 27 | CH22 |
| 02-018 | 41 | CH23 |
| 03-001 | 4 | CH24 |
| 03-002 | 8 | CH25 |
| 03-004 | 19 | CH26 |
| 03-005 | 23 | CH27 |
| 03-006 | 30 | CH28 |
