# Supplementary material for: CDK4/6 inhibition in advanced chordoma: final results of the NCT PMO-1601 trial
Source: ESMO Open. 2025 Jul 7;10(7):105498. doi: 10.1016/j.esmoop.2025.105498 (PMC12272896; doi:10.1016/j.esmoop.2025.105498)
Supplement: Supplementary Table 5-6 [file mmc5.docx]

**Table S5. Clinical Parameters and impact on median PFS and median OS**

|  | **Ki67* low<10 %**  n=22 | **Ki67 high ≥10 %**  n=5 |  |
| --- | --- | --- | --- |
| mPFS | 5.55 months | 8.1 months | **p=0.67**  HR 0.8, 95% CI 0.27, 2.35 |
| mOS | 24.6 months | 11 months | **p=0.48**  HR 0.66, 95% CI 1.8, 2.43 |
|  | **Therapy naïve**  n=7 | **Pre-treated**  n=21 |  |
| mPFS | 9.1 months | 5.52 months | **p=0.73**  HR 0.8, 95% CI 0.32, 2.22 |
| mOS | 17 months | 34 months | **p=0.91**  HR 0.92, 95% CI 0.21, 3.95 |
|  | **Age <60 years**  n=16 | **Age ≥60 years**  n=12 |  |
| mPFS | 5.67 months | 4.29 months | **p=0.53**  HR 0.77, 95% CI 0.32, 1.81 |
| mOS | 34 months | 24.5 months | **0.59**  HR 1.3, 95% CI 0.4, 3.77 |
|  | **IHC score CDK4**  **0-1**  n=17 | **IHC score CDK4**  **2-3**  n=11 |  |
| mPFS | 5.4 months | 9.1 months | **p=0.16**  HR 1.8, 95% CI 0.78, 4.16 |
| mOS | 34 months | 33 months | **p=0.57**  HR 1.36, 95% CI 0.43, 4.32 |
|  | **IHC score CDK6**  **0-1**  n=16 | **IHC score CDK6**  **2-3**  n=12 |  |
| mPFS | 5.55. months | 5.55 months | **p=0.95**  HR 1, 95% CI 0.44, 2.4 |
| mOS | 24.6 months | 47 months | **p=0.39**  HR 1.5, 95% CI 0.53, 4.33 |
|  | **IHC score CDK4**  **and CDK6 0-1**  n=24 | **IHC score CDK4**  **and CDK6 2-3**  n=4 |  |
| mPFS | 5.55 months | 8.32 months | **p=0.89**  HR 0.93, 95% CI 0.3, 2.82 |
| mOS | 34 months | 18.2 months | **p=0.84**  HR 0.86, 95% CI 0.17, 4.19 |
|  | **IHC Score Rb/pRB 0-2****  n=8 | **IHC Score Rb/pRb 3**  n=20 |  |
| mPFS | 11 months | 5.54 months | **p=0.26**  HR 1.7, 95% CI 0.7, 4.2 |
| mOS | not reached | 24.6 months | **p=0.87**  HR 0.9, 95% CI 0.26, 3.14 |

**mPFS** median progression free survival; **mOS** median overall survival

**Test used: Log rank test**

*****One missing data

******Only 1 patient with Rb/pRB score 0-1

**Table S6**. **Treatment related adverse events summary**

| **TRAE** | **Patients (n=28)** |
| --- | --- |
| TRAE, any grade | 27 (96.4%) |
| Grade 3 TRAE | 11 (39.2%) |
| Grade 4 TRAE | 0 |
| Fatal TRAE | 1 (3.6%) |
| TRAE leading to temporarily dose interruption/reduction | 8 (28.6%) |
| TRAE leading to treatment discontinuation | 1 (3.6%) |

**TRAE** Treatment Related Adverse Events
