# Supplementary material for: CDK4/6 inhibition in advanced chordoma: final results of the NCT PMO-1601 trial
Source: ESMO Open. 2025 Jul 7;10(7):105498. doi: 10.1016/j.esmoop.2025.105498 (PMC12272896; doi:10.1016/j.esmoop.2025.105498)
Supplement: Supplementary Table 7 [file mmc6.docx]

**Table S7. *CDKN2A* alterations and mRNA Expressions**

***CDKN2A* genomic alterations, mRNA expression and immunohistochemistry**

| **ID** | ***CDKN2A* CNA** | ***CDKN2A***  **SNV** | ***CDKN2A* mRNA log_2_(TPM+0.01)** | **CDKN2A (p16) IHC** | **Response after 6 cycles** |
| --- | --- | --- | --- | --- | --- |
| CH01 | Homozygous deletion | no | 0.22650853 | negative | PD |
| CH02 | Homozygous deletion | no | 0.992768431 | negative | PD |
| CH03 | Focal homozygous deletion | no | 0.422233001 | negative | PD |
| CH04 | Focal homozygous deletion | no | Not available | negative | PD |
| CH05 | Focal homozygous deletion | no | Not available | negative | PD |
| CH07 | Focal homozygous deletion, subclonal | H98P | Not available | negative | SD |
| CH08 | Heterozygous deletion | S12X | 4.524189078 | negative | SD |
| CH09 | Heterozygous deletion | no | Not available | negative | PD |
| CH10 | Heterozygous deletion | no | 3.116031993 | negative | SD |
| CH11 | Focal CDKN2A deletion Intron 1-2, heterozygous | no | 2.918386234 | negative | PD |
| CH12 | Focal homozygous deletion | no | 1.790772038 | negative | SD |
| CH13 | Focal loss, homozygous deletion | no | Not available | negative | PD |

**CNA** Copy Number Alteration; **TPM** Transcript Per Million; **IHC** Immunohistochemistry

**mRNA and immunohistochemistry-based expression (*CDK4, CDK6, CCND1, RB1*)**

| **ID** | **CDK4 mRNA**  **log2(TPM+0.01)** | **CDK4**  **IHC grade** | ***p**** | **CDK6 mRNA**  **log2(TPM+0.01)** | **CDK6**  **IHC grade** | ***p**** | **CCND1 mRNA**  **log2(TPM+0.01)** | **CCND1****  **IHC grade** | **RB1 mRNA*****  **log2(TPM+0.01)** | **RB1/pRBS780**  **IHC grade** |
| --- | --- | --- | --- | --- | --- | --- | --- | --- | --- | --- |
| CH01 | 6.721235931 | 1 | 0.69 | 4.567423758 | 3 | 0.53 | 7.983050597 | n.a. | 4.931210275 | 3 |
| CH02 | 6.472650228 | 0 |  | 3.820689561 | 3 |  | 7.614783449 | n.a. | 4.130107179 | 3 |
| CH03 | 6.930855586 | 0 |  | 3.44625623 | 0 |  | 7.409475798 | n.a. | 4.088311236 | 1 |
| CH08 | 6.728056551 | 3 |  | 4.170726276 | 1 |  | 6.487196907 | 0 | 4.644433152 | 3 |
| CH10 | 6.830990481 | 1 |  | 3.820689561 | 1 |  | 7.530211233 | 3 | 4.46008724 | 2 |
| CH11 | 6.728056551 | 3 |  | 4.700994494 | 3 |  | 7.930796463 | 3 | 5.49217366 | 2 |
| CH12 | 6.436461703 | 1 |  | 3.323370069 | 3 |  | 6.644000452 | 2 | 4.000901403 | 3 |

Non-parametric Wilcoxon rank test, *p<0.05 is considered significant (IHC 0, 1+ ***vs*** IHC 3+

**correlation test between mRNA expression and protein expression (IHC score) was not possible due to low numbers

***correlation between mRNA and IHC score was not performed because the score also includes the pRBS780 not reflected by mRNA-seq

**n.a**. – not available

**Correlation between CDKN2A mRNA expression according to zygosity**

| ***CDKN2A* heterozygous deletion** | ***CDKN2A* homozygous deletion** |
| --- | --- |
| 3.116031993 | 0.22650853 |
| 2.918386234 | 0.992768431 |
| 4.524189078 | 0.422233001 |
|  | 0.22650853 |
| **p=0.034*** | |

Unpaired t-test, two-sided with Welch correction, p<0.05 is considered significant

*Non-parametric Wilcoxon rank test, p<0.05 is considered significant
