# Supplementary material for: CDK4/6 inhibition in advanced chordoma: final results of the NCT PMO-1601 trial
Source: ESMO Open. 2025 Jul 7;10(7):105498. doi: 10.1016/j.esmoop.2025.105498 (PMC12272896; doi:10.1016/j.esmoop.2025.105498)
Supplement: Supplementary Information [file mmc7.docx]

**Trial population**

**Inclusion Criteria**

1. Patients with locally advanced or metastatic chordoma with confirmed diagnosis in a reference pathology (with immunohistology for epithelial membrane antigen, S100, Brachyury, INI-1) not amenable to curative treatment with surgery or radiotherapy.

2. At least one measurable tumor lesion according to RECIST 1.1 criteria

3. Loss of p16 determined immunohistochemically or CDKN2A/B genomically, presence of CDK4/6 and RB1 determined immunohistochemically or by RNA sequencing.

4. Age ≥ 18 years, no upper age limit

5. Availability of tissue blocks preferably not older than 12 months for immunohistologic assessment (if no adequate material is available, re-biopsy should be considered before entering the study)

6. Non-pregnant and non-nursing. Women of child-bearing potential must have a negative serum or urine pregnancy test with a sensitivity of at least 25 mIU/mL within 72 hours prior to registration (WOCBP is defined as a sexually active mature woman who has not undergone a hysterectomy or who has had menses at any time in the preceding 24 months).

7. Women of child-bearing potential must either commit to continued abstinence from heterosexual intercourse or use a highly effective method of birth control (e.g. double barrier contraceptive method (IUD, condome), tubal ligation, or partner’s vasectomy) while on therapy and for 14 weeks after the last dose of therapy. Hormonal contraception alone is an inadequate method of birth control. Female patients must agree not to donate lactation during treatment and until 14 weeks after end of treatment.

8. Men must agree not to father a child and must use a latex condom during any sexual contact with WOCBP while receiving therapy and for 14 weeks after therapy is stopped, even if they have undergone successful vasectomy. Sperm donation is not permitted for the same time interval.

9. Signed written informed consent

10. Performance status ≤ 2 according to ECOG/WHO criteria

11. Ability of patient to understand the character and individual consequences of clinical trial

**Exclusion Criteria**

1. Prior treatment with palbociclib or known intolerance/allergy to the compound or any ingredient (acquired or hereditary).

2. Prior treatment with other CDK4/6 inhibitors

3. Co-therapy with strong/potent CYP3A inducers and/or inhibitors, (e.g., clarithromycin, indinavir, itraconazol, ketoconazol, lopinavir/ritonavir, nefazodon, nelfinavir, posaconazol, saquinavir, telaprevir, telithromycin, voriconazol, and St. John’s Wort [Hypericum perforatum]) while on treatment with study drug

4. Co-therapy with corticosteroids above 7.5 mg prednisolone/prednisone equivalent

5. Anticancer treatment less than 2 weeks prior to study treatment

6. Organ insufficiency: creatinine clearance <30 ml/min; total bilirubin >1.5x upper normal serum level; AST > upper normal serum level; abnormal blood counts; heart failure (New York Heart Association (NYHA) III/IV); uncontrolled hypertension; unstable angina; serious cardiac arrhythmia; severe obstructive or restrictive ventilation disorder.

7. Clinical signs of active infection (>Grade 2 according to CTCAE version 5.0)

8. Patients with a “currently active” second malignancy other than non-melanoma skin cancer. Patients are not considered to have a “currently active” malignancy if they have completed therapy and are considered by their physician to be at less than 30% risk of relapse within one year.

9. Severe neurologic or psychiatric disorder interfering with ability of giving informed consent

10. Known or suspected active alcohol or drug abuse

11. Known positivity for HIV, active HAV, HBV, or HCV infection

12. Cytopenia: platelets <100 G/l, neutrophils <1.0 G/l, hemoglobin <10.0 g/dl

13. Corrected QT interval (QTcB) >470 msec (based on the mean value of triplicate ECGs), family or personal history of long or short QT syndrome, Brugada syndrome, or known history of QTcB prolongation or Torsade de Pointes

14. Uncontrolled electrolyte disorders that can aggravate the effects of a QTcB-prolonging drug (e.g., hypocalcemia, hypokalemia, hypomagnesemia)

15. Participation in other ongoing interventional clinical trials (according to AMG) within 4 weeks prior to study treatment.
