# Supplementary material for: CDK4/6 inhibition in advanced chordoma: final results of the NCT PMO-1601 trial
Source: ESMO Open. 2025 Jul 7;10(7):105498. doi: 10.1016/j.esmoop.2025.105498 (PMC12272896; doi:10.1016/j.esmoop.2025.105498)
Supplement: Supplementary Data [file mmc8.pdf]

Supplementary figures

Figure S1

A

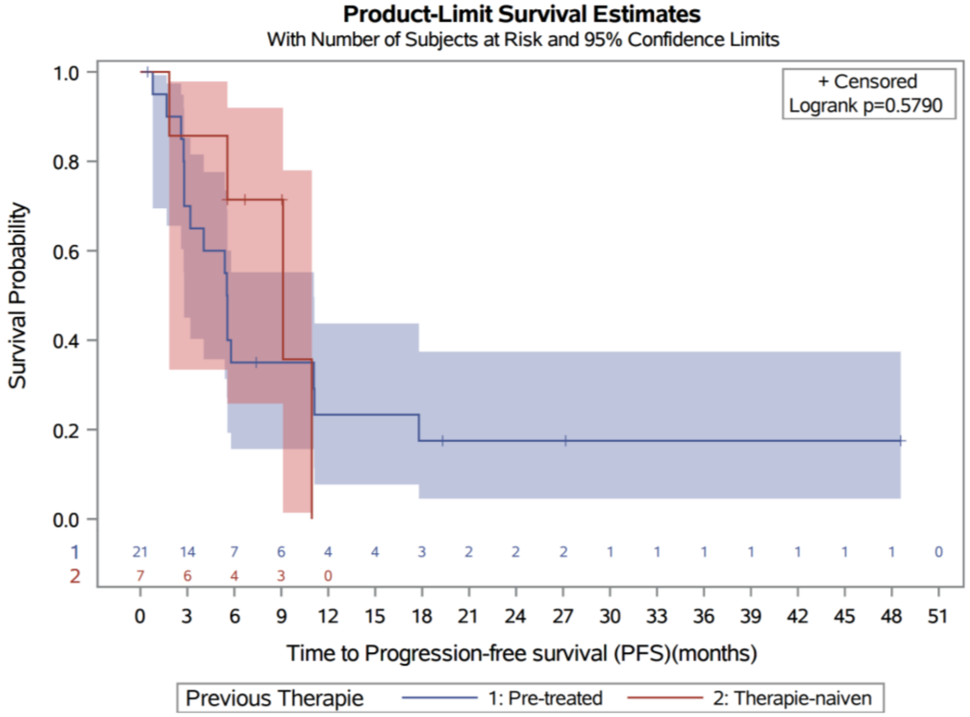

B

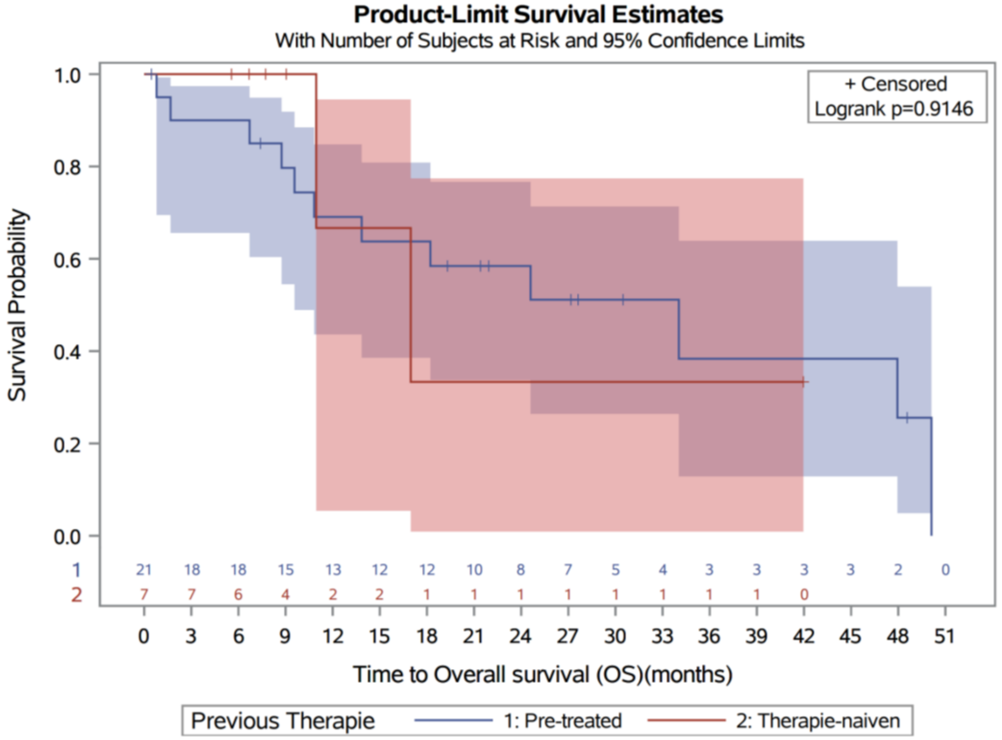

Figure S2

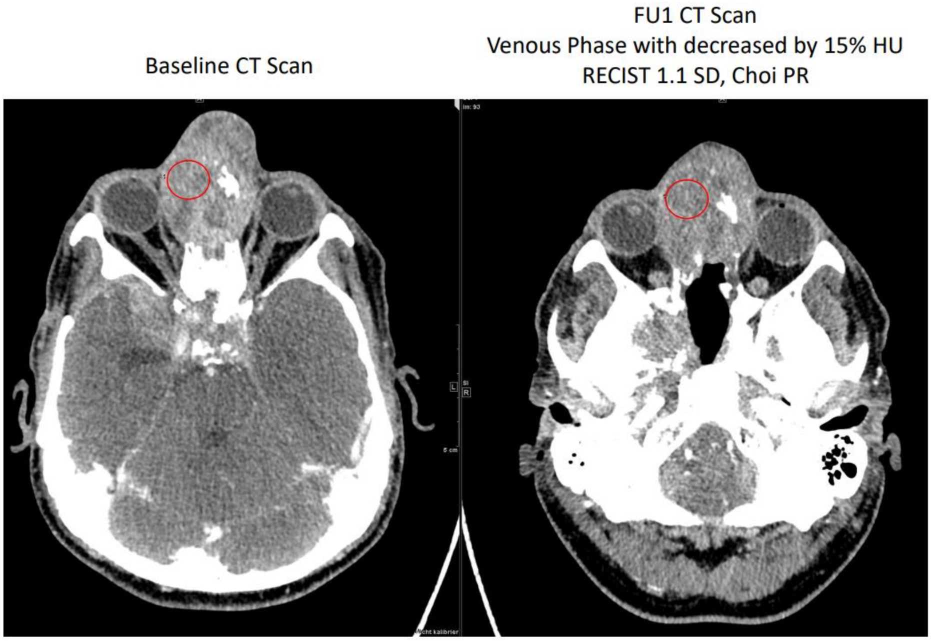

Figure S3

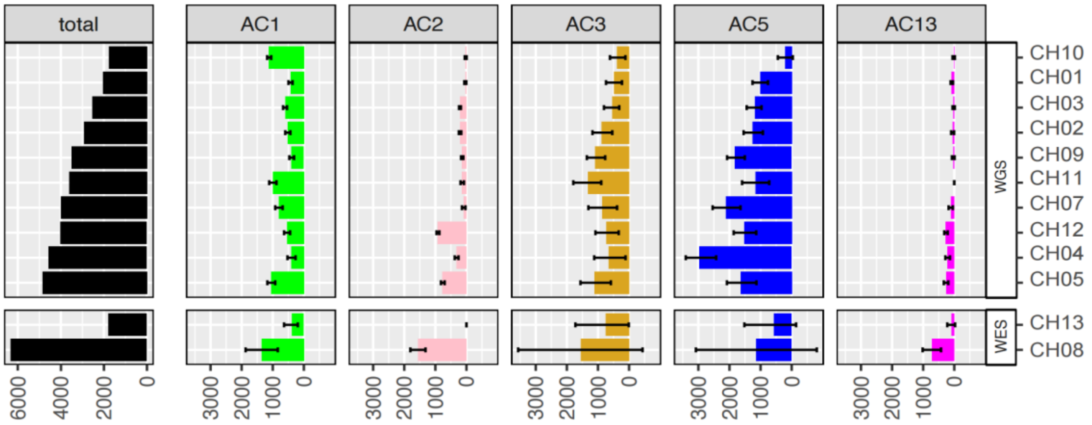

Figure S4

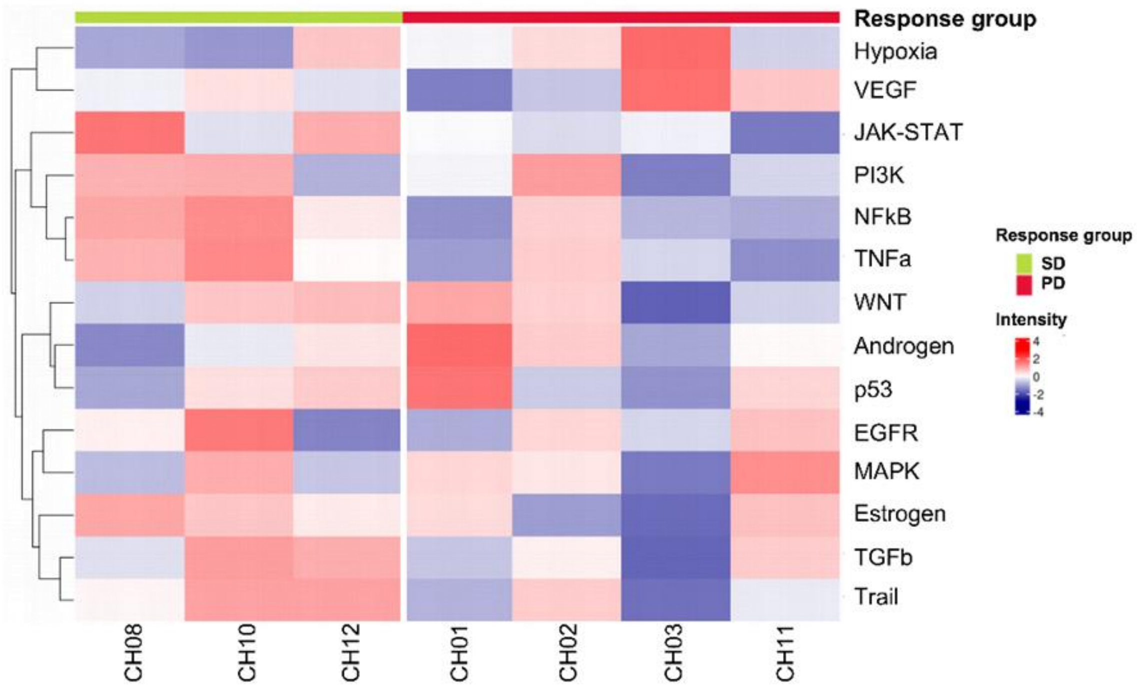

Figure S5

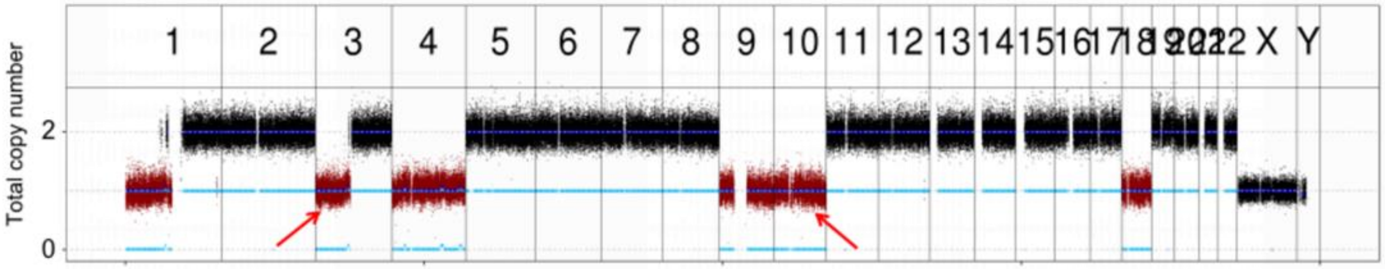

Supplementary figures descriptions

Figure S1. Survival analysis. A. Progression free survival in the safety population for pre-treated patients vs treatment naïve patients. Median (95% Confidence Interval (lower, upper)) for pre-treated patients 5.5 (2.8, 11.1) months vs 9.1 months (1.8, .) for treatment naïve patients. p=0.57. **B.** Overall survival in the safety population for pre-treated patients vs treatment naïve patients. Median (95% Confidence Interval (lower, upper)) for pre-treated patients 34.0 (9.6, .) months vs 17.0 months (11.0, .) for treatment naïve patients. p=0.91.

Figure S2. CT scan showing a partial response by Choi criteria in a patient with clivus chordoma (CH09) with stable disease per RECIST at 3 months assessment. **FU1** – follow-up 1, **PR**-partial response, **SD**-stable disease

Figure S3. Mutational signatures showing APOBEC (AC2 and AC13), AC3 and AC5 as the most prevalent signatures.

Figure S4. Pathway activity in seven chordoma patients with available RNA-seq data using the PROGENy algorithm. Several distinct pathways are deregulated at study entry. Patients are grouped according to response status after six cycles in SD and PD. **SD**, stable disease; **PD** progressive disease

Figure S5. Copy number plot by WGS illustrating a diploid tumor with heterozygous loss of chromosomes 3p and 10q, where *VHL* and *PTEN* are located.
